# Pacemaker Failure to Capture Caused by Electrocautery: A Rare Pacemaker Pulse Generator Change Complication

**DOI:** 10.7759/cureus.28252

**Published:** 2022-08-22

**Authors:** Aman Qureshi, Intisar Ahmed, Aamir H Khan

**Affiliations:** 1 Research, Aga Khan University, Karachi, PAK; 2 Cardiology, Department of Medicine, Aga Khan University, Karachi, PAK

**Keywords:** complication, failure to capture, electrocautery, generator change, pacemaker

## Abstract

In the advent of increasing benefits of cardiac devices, more and more implants are being done. Pacing devices reaching the end of service need to be changed. The use of electrocautery (EC) to maintain hemostasis during cardiac device implantation is efficient and safe. Device makers have variable recommendations for the use of EC. Generally, considered safe, EC has been rarely known to cause device failure. We describe a case of a dual-chamber device, pulse generator change, where EC caused a sudden, unexpected loss of pacing function that lasted for 30 seconds. This case report highlights the gaps in the process of undertaking these high-risk changes.

## Introduction

In the 21st century, more and more devices are being implanted, and these have shown benefits for quality of life and in some cases mortality [1,2]. The limitation, however, remains the battery longevity, with most pacemakers (PMs) rarely going over 10-12 years. After the device reaches the elective replacement indication (ERI) or the end of the service period, the generator needs to be replaced, although three to six months of battery longevity remains [3]. Currently, it is recommended to change the device within three months of achieving ERI; the pacemaker (PM) is deemed fit in this period, albeit with some curtailment of function [4].

Electrocautery (EC), both unipolar and bipolar, is often used in the procedure for the replacement of the generator [5]. In the ERI period, the use of EC is considered safe; however, it is recommended that very short bursts of a lower amplitude are used, and careful continuous rhythm monitoring is pursued. Rarely, device malfunction related to cautery has been reported [6]. We describe a case where a dual-chamber pacemaker generator was being changed and resulted in a failure to capture due to electromagnetic interference (EMI) from the EC. The case highlights a rare complication and emphasizes the use of closed-loop communication in the electrophysiology (EP) laboratory.

## Case presentation

An 88-year-old male was seen in the clinic. He had a dual-chamber pacemaker Identity^TM^ ADx XL DR (St. Jude Medical, Saint Paul, Minnesota, USA) implanted in 2013 for complete atrioventricular (AV) block with slow ventricular escape rhythm. In the pacemaker clinic, the device was noted to be in the ERI period mandating a change. The leads were checked for integrity and both atrial sensing, pacing, and impedance values were within normal range and showed a steady chronic trend. The patient had no underlying ventricular rhythm and was in an atrial sense (AS) and ventricular pace (VP) configuration. The device had reset the pacing output to unipolar.

**Figure 1:**
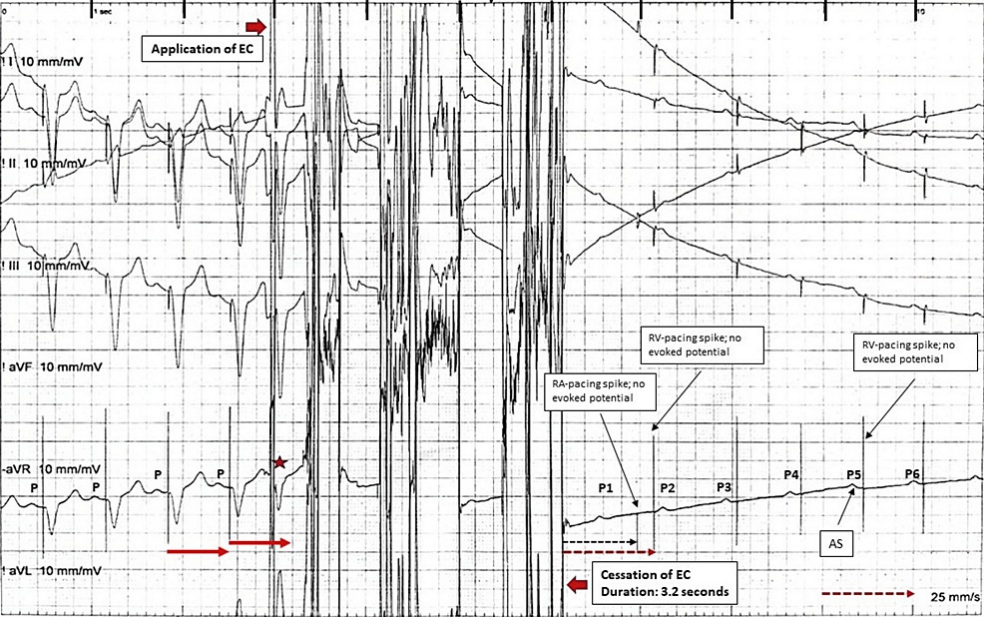
Electrocardiogram During Electrocautery Six-lead rhythm strip that shows atrial sensing, followed by a unipolar pacing spike and appropriate ventricular pacing. There is an application of electrocautery (EC) for a period of 3.2 seconds and then an abrupt failure to pace. The first beat after the application of EC (star) is possibly a tracked-paced beat. After EC cessation, the first two P waves (P1 and P2) are not sensed. There are atrial and ventricular pacing spikes without evoked potentials (failure to pace). Onward from P4, there is appropriate atrial sensing then the programmed AV delay and unipolar ventricular pacing spikes, but no ventricular evoked potential (failure to pace), which remains for the duration of the trace.

**Figure 2:**
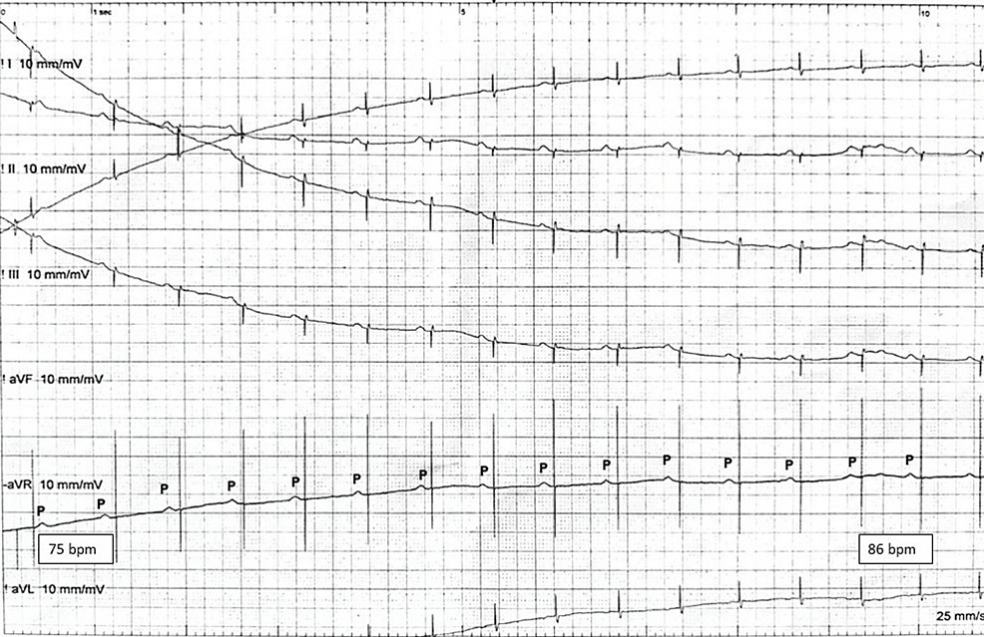
Six-Lead Electrocardiogram The figure shows the continuation of the six leads from Figure 1, showing atrial sensing and ventricular pacing spike with failure to capture. The device outputs after the programmed AV delay but does not capture the myocardium. The sinus rate goes from 75 to 86 bpm in 10 seconds.

On the day of the procedure, the patient was hemodynamically stable with preliminary laboratory workup all within the normal range. Manually operated transcutaneous pacemaker (TCP) pads were applied that are used for both cardioversion/defibrillation and pacing. The return patch for the EC was placed on the right leg. Although the patient was pacemaker-dependent, we did not put a temporary pacemaker as it increases the risk of infection. After preparing the left infraclavicular area, midazolam 1 mg aliquots to a total of 3 mg were administered to establish conscious sedation. The old scar was removed using an ellipsoid wedge incision. To stop bleeding in the scar, unipolar EC was used in a short burst. The EC was set at 45 watts, and only coagulation was used. This resulted in a sudden and unexpected loss of ventricular capture (Figure 1 and Figure 2).

The loss of capture (LOC) remained for 30 seconds, and before the TCP could kick in, the ventricular capture through the permanent pacemaker spontaneously resumed. The further procedure was done using blunt dissection. When the device was removed from the pocket, a few beats were missed because of unipolar pacing, necessitating the generator to be repositioned in the pocket. The device was replaced into the pocket, and pacing resumed. The device ventricular lead was then carefully shifted to the analyzer cable, and subsequent pacing was done through it. The leads were rechecked for thresholds and impedance and were found in the normal range. The new device Endurity MRI DR (Abbott, Chicago, Illinois, USA) was attached and tested. At the moment of LOC, the device was deep in the pocket, as only the incision had taken place, the cautery tip was away from the device, and there was no direct contact. After the resumption of pacing, the patient’s recovery was uneventful; he woke up to tactile stimulus and did not experience symptoms of cerebral hypoperfusion. The patient was discharged the next day and was followed up in the clinic a week after the implant and was doing well.

Following the case, the team took part in debriefing, and it was unanimously felt that closed-loop communication was lacking, and unipolar pacing noted in the clinic was not confirmed in the laboratory. It was assumed that the mode was asynchronous, and the ventricular polarity was bipolar (less prone to electromagnetic interference). This happened as the team did not practice closed-loop communication, despite a call for a time out. Femoral access for quick temporary pacemaker (TPM) insertion would have been a prudent step for a pacemaker-dependent case.

## Discussion

Pacemaker generator change and the use of EC are common. It is classic teaching not to use EC over the device, and this recommendation came out in the 1980s for older devices [7]. Current experience suggests that surgical EC causes little or no damage to current device systems [4]. The procedure is usually recommended to be done in the dual-chamber asynchronous pacing (DOO), and as mostly bipolar leads are used, electromagnetic interference (EMI) by EC becomes less likely. Even when it is in a dual-chamber (DDD) mode, short-burst EC in a ratio of 1:10 seconds (application of EC for one second and a waiting period of 10 seconds) is considered safe [8]. The recommendation from the major device makers varies from no EC to no direct contact of EC with the device or lead system [4]. Even when there is noise detection in the noise sampling period of the ventricular lead, there is reset, and with short bursts as described, at most one beat may be missed [9]. In our case, however, there was a failure to pace in the ventricle that lasted 30 seconds, which was unexpected. The patient did not experience hypoperfusion seizures possibly because of sinus rhythm (atrial kick, which provides 20%-30% of the cardiac output), providing some cardiac output [10]. As the patient was supine, hemodynamics was better tolerated.

In literature, asymptomatic patients with sinus rhythm, in the awake upright position with more than 10 seconds of ventricular standstill, have been described; common among them is sinus rhythm as against asystole [11]. EC can interact with the device in myriad ways; it may cause sudden discharge and failure to output, it may track the EC signal in the atria and pace the ventricle at the upper track rate, and it may reset to a ventricular asynchronous pacing (VOO) or DOO setting, or it may cause noise reversion and asynchronous pacing [12].

Transient suppression of PM by EC is not uncommon, but prolonged failure to capture, as in our case, has been reported earlier but is a rare finding [4,13,14]. Mangar et al. described the case of a 15-year-old female undergoing surgery who had her PM (ventricular demand pacing (VVI)) switched to asynchronous mode but experienced asystole with the application of unipolar EC, which caused decreased device voltage and failure [13]. Asynchronous modes, believed to protect against over-sensing, are not protected from sudden battery discharge [4]. Abdelmalak et al. described a case of a 74-year-old male undergoing a thoracic laminectomy procedure; the PM was in DDD mode, and the application of coagulation EC caused transient asystole, which recovered immediately after the cessation of application. This issue was circumvented when they used cutting EC. Modulated signals are used for cutting EC using bursts of energy versus coagulation EC where unmodulated signals heat the tissue [14]. Nagarakanti et al. described two cases. One was a 74-year-old male who was undergoing a cardiac resynchronization defibrillator (CTR-D) generator change during the ERI period. The mode was set to VOO and all leads to bipolar configuration. A sharp incision followed by EC was performed for maintaining homeostasis, which was stopped prior to reaching the device. On removing the device from the pocket, the device stopped pacing and did not resume pacing after repositioning back in the pocket. Emergent unipolar pacing through the device set screw using a hex wrench restarted pacing. The second case was a 79-year-old male who had a St. Jude Integrity DR pacemaker and was changed before ERI due to travel reasons. The pacemaker mode was programmed to VOO, as the patient had no underlying rhythm. During dissection to free the device from adhesions using EC, the heart rate decreased to 30 bpm, which was half of the programmed rate. The lead was detached, and the pacing was done through the lead analyzer. The leads later tested were found to be normal [4].

**Figure 3:**
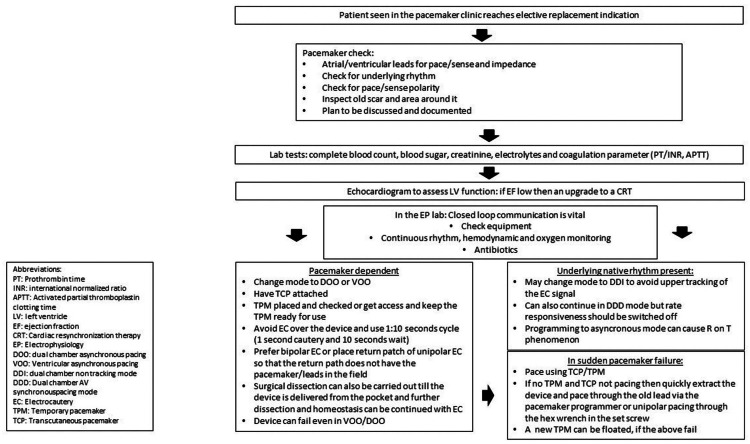
Algorithm for Pacemaker Pulse Generator Change

ERI is a defined period in which the pacemaker performs all normal functions with some restrictions to some diagnostic and rate responsive features. It is recommended that the generator should be changed in this period. In our case, we were well within the ERI period, and this failure was not expected. We learned that a DOO/VOO mode in a bipolar configuration, with a standby temporary pacing wire, or a TPM in place is a prudent strategy for a pacemaker-dependent patient. However, the cases described above show us that the sudden and unexpected loss of pacing is possible, albeit rare. A good EP laboratory would be prepared for this catastrophe, only if the team keeps an index of suspicion. Both in our case and the first case of Nagarakanti et al., the EC tip had not come in direct contact with the device, yet it failed to pace. Ideally, one should use bipolar EC, but if one must use unipolar EC, the return or dispersive pad should be placed in a way to keep the device out of the field of the EC. If that cannot be done, then reliance should be on surgical dissection. Learning from our experience, we suggest following a standard algorithm to minimize unexpected complications (Figure 3).

## Conclusions

Although electrocautery is an effective tool for achieving hemostasis during pacemaker pulse generator change, it interferes with a pacemaker and may result in potentially life-threatening situations. Detailed interrogation of a pacemaker, including remaining battery voltage and pace/sense polarities, is recommended before starting the procedure. Switching a pacemaker to asynchronous mode minimizes electromagnetic interference-induced over-sensing, and it does not prevent the effect of electrocautery on pacing function. Closed-loop communication and dedicated algorithms are required to prevent these complications in the electrophysiology laboratory.
